# Evaluation of the performance of tests for spatial randomness on prostate cancer data

**DOI:** 10.1186/1476-072X-8-41

**Published:** 2009-07-03

**Authors:** Virginia L Hinrichsen, Ann C Klassen, Changhong Song, Martin Kulldorff

**Affiliations:** 1 Department of Ambulatory Care and Prevention, Harvard Medical School and Harvard Pilgrim Health Care, Boston, MA 02215, USA; 2 Department of Health, Behavior and Society, Johns Hopkins Bloomberg School of Public Health, Baltimore, MD 21205, USA; 3 Department of Statistics, University of Connecticut, Storrs, CT 06269, USA

## Abstract

**Background:**

Spatial global clustering tests can be used to evaluate the geographical distribution of health outcomes. The power of several of these tests has been evaluated and compared using simulated data, but their performance using real unadjusted data and data adjusted for individual- and area-level covariates has not been reported previously.

We evaluated data on prostate cancer histologic tumor grade and stage of disease at diagnosis for incident cases of prostate cancer reported to the Maryland Cancer Registry during 1992–1997. We analyzed unadjusted data as well as expected counts from models that were adjusted for individual-level covariates (race, age and year of diagnosis) and area-level covariates (census block group median household income and a county-level socioeconomic index). We chose 3 spatial clustering tests that are commonly used to evaluate the geographic distribution of disease: Cuzick-Edwards' *k*-NN (*k*-Nearest Neighbors) test, Moran's I and Tango's MEET (Maximized Excess Events Test).

**Results:**

For both grade and stage at diagnosis, we found that Cuzick-Edwards' *k*-NN and Moran's I were very sensitive to the percent of population parameter selected. For stage at diagnosis, all three tests showed that the models with individual- and area-level adjustments reduced clustering the most, but did not reduce it entirely.

**Conclusion:**

Based on this specific example, results suggest that these tests provide useful tools for evaluating spatial clustering of disease characteristics, both before and after consideration of covariates.

## Background

Spatial clustering tests are often used to determine whether health events are geographically clustered or whether they are distributed randomly throughout space as expected by chance. When clustering exists in the data, it is typically caused by geographic variation in disease risk factors, which may include characteristics of individuals, environmental influences on disease, or health care services which may serve to influence the distribution of disease characteristics of interest. For example, without age adjustment, crude or unadjusted cancer incidence rates vary geographically, as the risk for most cancers increases with age, and age distributions in most populations vary geographically. Lung cancer rates may vary geographically because of geographical differences in smoking habits and certain occupational exposures, while the proportion of late stage breast cancer may vary geographically because of geographical differences in access to mammography screening programs. Examples of clusters identified in previous work are high prevalence gonorrhea transmission areas [1], census tracts with significantly high proportions of men diagnosed with distant-stage prostate cancer [2], cases of acute lymphoblastic leukaemia in Great Britain [3], health regions with a high incidence of liver cancer in Ontario, Canada [4] and breast cancer deaths in the New York City – Philadelphia metropolitan area [5]. When studying the geographical distribution of disease, analyses are almost always adjusted for age and gender, as we are interested in geographical variation that is not explained by these two factors. If there is still spatial clustering in the data after such adjustments, there are other disease risk factors that are unevenly distributed geographically.

There are two types of cluster detection: local or "hot-spot" tests, and tests of global clustering. Hot-spot cluster detection tests identify and evaluate specific local clusters, which may be of interest to investigate specific local causes of such clusters, or to target service delivery to higher need areas. Global clustering tests are used to test whether clustering exists as a general phenomenon in the study region, and thus can be used to answer more general questions regarding variation in the distribution of disease characteristics, and propensity of disease characteristics to cluster geographically. For certain public health questions, this may identify an infectious or communicable aspect of a disease process. For non-communicable diseases, assessing the degree of clustering in regard to disease characteristics of interest may inform researchers or service providers about variation in rates of disease risk or detection across a given geographic area [6,7].

If there are statistically significant hot-spot clusters, we may search for such additional risk factors in the location of the hot-spot cluster. If there is statistically significant global clustering, we may search for additional risk factors among variables of a more global nature. If there is no statistically significant clustering, we may search for additional risk factors that are more evenly distributed geographically.

As an illustrative example of how global clustering tests perform when evaluating residual spatial clustering, we used prostate cancer data from the Maryland Cancer Registry, where registry data are mostly complete and previous work has been done on modeling risk factors for higher grade and later stage at diagnosis. Prostate cancer is the most common diagnosed cancer among men in the US, representing 29% of incident cancer cases expected to occur among men in 2007 [8]. It is estimated that the vast majority of these cases will be diagnosed at stages where 5-year relative survival is near 100% [8]. However, among cancer deaths reported from 2004, prostate cancer is the second leading cause of cancer death in men in the US [8]. Prostate cancer disease characteristics and treatment vary by a number of characteristics, including socioeconomic status [9], race [10] and geography [10,11]. Area-level measures of socioeconomic status have not been shown to explain racial disparities in prostate cancer [12].

Prior studies have shown that geographical clustering can be reduced or eliminated by adjusting for individual-level covariates [13-16] and by incorporating random effects into models [4,11,17,18]. Joint spatial survival models of prostate cancer age at diagnosis and survival [19] and Bayesian hierarchical models of prostate cancer stage at diagnosis [18] have been used to investigate spatially clustered patterns. These studies show that factors related to individuals and their communities likely contribute to disease clustering. They demonstrate that once clustering is identified by a clustering test, further evaluation of other predictors of disease can be important to further investigate the risk.

Our current study further examines geographical clustering by evaluating the performance of 3 global clustering tests on prostate cancer data adjusted for both individual-level and area-level covariates. We selected 3 global clustering tests which use frequentist methods of inference: Cuzick-Edwards' *k*-NN [20], Moran's I [21] and Tango's Maximized Excess Events Test (MEET) [22]. Prior studies have examined the performance of these tests on simulated data [23,24]. The current study evaluates the performance of each test on real data on prostate cancer, from a large population-based cancer registry, adjusted for variables shown to be associated with prostate cancer grade and stage at diagnosis [10], to examine and illustrate the advantages and disadvantages of these methods. Additionally, using each test, we evaluate whether adjusting for individual- and/or area-level variables eliminates geographical clustering of prostate cancer grade and stage at diagnosis.

## Methods

**Figure 1:**
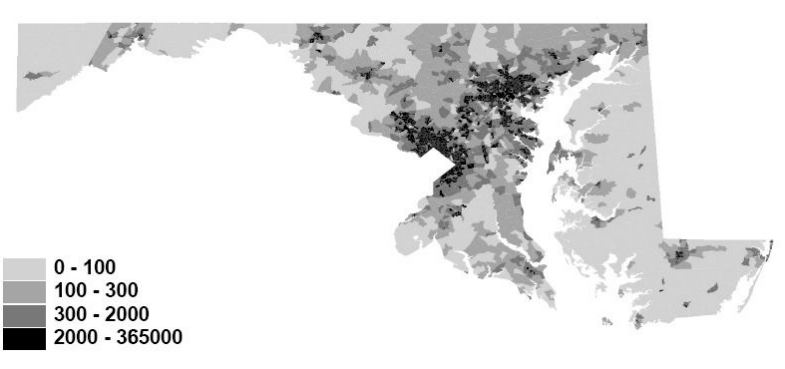
**Maryland Population Density – 1990 Census – Population per Square Mile by Census Block Group**.

In our previous work, we examined predictors of prostate cancer histologic tumor grade and stage of disease at diagnosis among incident cases reported to the Maryland Cancer Registry during 1992–1997. We dichotomized the outcomes as stage 1 versus stages 2,3,4,5 and 7 ("later stage"), and grades 1 and 2 versus 3 and 4 ("higher grade"), representing one of several possible clinically meaningful cutpoints for dichotomization. Figure 1 shows the Maryland population density by block group based on 1990 census data. Maps of the proportion of higher grade (grades 3 and 4) and later stage (stages 2 through 7) cases at diagnosis are available at: 

Methods for assigning geographic location and area-level covariates to cases are described in detail elsewhere [17]. We geocoded all cases by street address, and used an imputation algorithm based on census population distribution within zip codes to assign location to non-geocoded cases.

In these analyses, for our dichotomized outcomes of higher grade and later stage at diagnosis, we examine unadjusted data (the ratio of block group-specific observed to expected cases) as well as expected counts from multivariate and multi-level binary logistic regression models that were adjusted for individual-level covariates (race, age and year of diagnosis) and both individual- and area-level covariates (census block group median household income and a county-level socioeconomic index). The choice of covariates used here was based on the most explanatory models for these outcomes in our previous work, and is explained in more detail elsewhere. The adjusted expected counts were used to calculate the block group-level expected counts for the four models (Table 1), as previously described [25]. Briefly, the logistic regression model with individual-level adjustments only included the following predictors of higher grade: older age, black race, and more recent year of diagnosis; and the following predictors of later stage: older age, black race, higher tumor grade, missing tumor grade, and more recent year of diagnosis. Models with individual-and area-level adjustments included the above predictors, as well as block group median household income and a county-level socioeconomic index (for higher grade) and block group percentage of white collar workers among those employed and a county-level socioeconomic index (for later stage). Two additional models were created for each outcome: one that included random intercept terms for both block group and county in the model with individual-level adjustments only, and one that similarly included random intercept terms for block group and county in the model with individual- and area-level adjustments. The inclusion of such terms changes the estimates of the covariates, and thus the expected counts in each block group. Multi-level models were estimated using the GLLAMM extension of STATA, and all modeling was done using STATA (STATA Corp, College Station, TX). We evaluated the performance of each of the three global clustering test statistics on the unadjusted data and on the expected counts from the four models.

**Table 1:** Data and models used to evaluate test statistics.

**Data/Model Number**	**Data/Model Description**
1	Unadjusted data

2	Individual-level adjustments; no area-level random effects

3	Individual-level adjustments; area-level random effects

4	Individual- and area-level adjustments; no area-level random effects

5	Individual- and area-level adjustments; area-level random effects

We used the following notation:

*c*_*i *_= the observed number of cases in block group *i*

*n*_*i *_= the expected cases in block group *i*

*H *= the total number of block groups

*d*_*ij *_= the Euclidean distance between block group *i *and *j*

*S*_*i*_*(k) *= the area in the smallest circle around *i *with at least *k *expected cases



The first test we evaluated was Cuzick-Edwards' *k*-NN (*k*-Nearest Neighbors) [20], which is defined as:



where *I *is the indicator function, such that *I*(true) = 1 and *I*(false) = 0.

Cuzick-Edwards' *k*-NN test was originally created to evaluate clusters in case-control data, but can easily be modified to handle aggregate data as well. We evaluated this test using 5 different parameter values. We defined *k*/N as a proportion of the population, where *k *is the number of cases, and used the following parameter values: *k*/N = 0.1%, 1%, 10%, 25% and 50%. Higher values of the test statistic indicate more clustering.

The next test we evaluated was Moran's I [21], which is defined as:



where *a*_*ij *_= 1 if *jεS*_*i*_*(k) *; 0 otherwise

Moran's I is a correlation test between nearest neighbors, originally designed to evaluate continuous data. Modifications to Moran's I have been used to assess spatial clustering, and in particular Local Moran's I is used as a local indicator of spatial correlation [26]. Moran's I is dependent on a weight function *a*_*ij*_. We define *a*_*ij *_to be 1 if the population (within the distance of block group *j *and block group *i*) is within a certain range. A distance-based proximity matrix also exists, but we did not evaluate it here. Results for Moran's I may differ depending on the chosen matrix. We evaluated this test by setting the range to each of the same 5 parameter values: *k*/N = 0.1%, 1%, 10%, 25% and 50%. Higher values of the test statistic indicate more clustering.

The final test we evaluated was Tango's MEET (Maximized Excess Events Test) [22], which is defined as:



where:



and



and *C*_*i *_is the random number of cases in block group *i *as generated by the null hypothesis.

Tango's MEET was designed to extend a general spatial clustering test to one that does not require specification of the scale parameter value λ, and thus avoids concerns with multiple testing if the same test is used more than once with different parameter values. Although this test uses a distance-based proximity matrix, tests with different distance parameters have been compared previously [23,27,28]. Smaller values of the test statistic indicate more clustering.

All three test statistics were implemented using Monte Carlo hypothesis testing [29], which is a randomized permutation based inference method that is commonly used in spatial statistics.

## Results

A total of 23,993 individuals were included in the population used for this analysis (Table 2). Approximately half were under 70 years of age, about three-quarters were white and one quarter was black. About 20% were later stage (stage 2, 3, 4, 5 or 7) when diagnosed, and about 20% were higher grade (grades 3 or 4) when diagnosed.

**Table 2:** Demographic characteristics of individuals included in the Registry.

	**Registry Population N = 23993**		**Stage Analyses N = 19223**		**Grade Analyses N = 18947**	
***Age Group***	n	%	n	%	n	%
16–49	403	2	352	2	325	2
50–69	11777	49	10228	53	9868	52
70–79	8739	36	6833	36	6853	36
80–106	3002	13	1810	9	1901	10
Missing	72	1	0	0	0	0
						
***Race***						
White	16565	69	14255	74	14114	74
Black	5779	24	4968	26	4833	26
Other	366	2	0	0	0	0
Missing	1283	5	0	0	0	0
						
***Stage at Diagnosis***						
0	80	1	0	0	0	0
1	15679	65	15233	79	13798	73
2	2250	9	2190	11	2000	10
3	263	1	255	1	220	1
4	170	1	165	1	152	1
5	150	1	145	1	127	1
7	1274	5	1235	7	945	5
Missing	4127	17	0	0	1705	9
						
***Grade at Diagnosis***						
1	2505	10	2042	10	2289	12
2	13112	55	11301	59	12335	65
3	4425	18	3786	20	4199	22
4	128	1	113	1	124	1
Missing	3823	16	1981	10	0	0

We found that Cuzick-Edwards' *k*-NN and Moran's I were very sensitive to the parameter selected for both grade and stage at diagnosis, with p-values ranging from 0.001 to 1.000 for the same method and data. The test performed most consistently when intermediate parameter values (1% and 10% of the population) were chosen rather than low or high parameter values (Table 3).

**Table 3:** Sensitivity of global clustering tests to the parameter chosen.

	Parameter (results reported as p-values)				
	0.10%	1%	10%	25%	50%

**Later Stage Cases (unadjusted data)**					

Cuzick-Edwards' *k*-NN	0.015	0.001	0.001	0.075	0.814

Moran's I	0.001	0.001	0.001	0.001	0.001

					

**Higher Grade Cases (unadjusted data)**					

Cuzick-Edwards' *k*-NN	0.738	0.001	0.001	0.002	0.045

Moran's I	0.044	0.001	0.001	0.005	1.000

To compare the performance of the clustering tests, we selected the Cuzick-Edwards' *k*-NN and Moran's I results using *k *= 1%. Tango's MEET does not require selection of a parameter value.

For prostate cancer stage at diagnosis, the models with individual- and area-level adjustments reduced clustering (i.e. explained that some of the spatial variation was due to these individual- and area-level influences) the most (Table 4). This was shown by all three tests. Of the two models with individual- and area-level adjustments, the one with no area-level random effects reduced clustering slightly more. The models with only individual-level adjustments also reduced clustering when compared to the unadjusted data. The additional area-level adjustments, however, further reduced the clustering.

**Table 4:** Global clustering test results with different adjustments.

**Data/Model**	**Cuzick-Edwards *k*-NN**		**Moran's I**		**Tango's MEET**	
	*k *= 1%		k= 1%			

	Test Statistic	p-value	Test Statistic	p-value	Test Statistic	p-value

**Later Stage**						

1:Unadjusted data	173544	0.001	0.779	0.001	<10^-15^	0.0001

2:Individual-level adjustments; no area-level random effects	170735	0.001	0.672	0.001	3.89 × 10^-15^	0.0001

3:Individual-level adjustments; area-level random effects	170899	0.001	0.675	0.001	3.44 × 10^-15^	0.0001

4:Individual- and area-level adjustments; no area-level random effects	168227	0.001	0.483	0.001	2.18 × 10^-08^	0.0001

5:Individual- and area-level adjustments; area-level random effects	168684	0.001	0.489	0.001	7.17 × 10^-10^	0.0001

						

**Higher Grade**						

1:Unadjusted data	197019	0.001	0.478	0.001	1.36 × 10^-13^	0.0001

2:Individual-level adjustments; no area-level random effects	195707	0.001	0.350	0.001	4.11 × 10^-08^	0.0001

3:Individual-level adjustments; area-level random effects	195727	0.001	0.351	0.001	4.31 × 10^-08^	0.0001

4:Individual- and area-level adjustments; no area-level random effects	195085	0.001	0.355	0.001	2.18 × 10^-08^	0.0001

5:Individual- and area-level adjustments; area-level random effects	197598	0.001	0.701	0.001	<10^-15^	0.0001

Higher values of the test statistic indicate more clustering for Cuzick-Edward's k-NN and Moran's I and less clustering for Tango's MEET. Test statistics can only be compared between models, not between the three methods.

For prostate cancer grade at diagnosis, models 2, 3 and 4 had consistent results across all tests, showing a reduction in clustering. However, the results from model 5 showed more clustering than in the unadjusted data, and this was a consistent finding across all tests.

## Discussion

We compared the performance of three global clustering tests on real unadjusted data, and data adjusted for variables potentially associated with prostate cancer grade and stage at diagnosis.

We found that the performance of Cuzick-Edwards' *k*-NN and Moran's I are sensitive to the parameter chosen by the user, and thus considered Tango's MEET the simplest test to use since it does not require selection of a scale parameter value. Its results were consistent with the results from Cuzick-Edwards' *k*-NN and Moran's I using an intermediate parameter value.

These statistical tests can be used to determine whether there is residual clustering after adjustments are made, and whether clustering is reduced or not by the adjustments. In this dataset we found that individual-level and area-level adjustments consistently reduced clustering in data on prostate cancer stage at diagnosis, but significant clustering remained. The results for prostate cancer grade were less consistent. It is possible that there are additional factors related to grade that we were not able to assess in these data. There are likely additional geographic elements that we did not account for that contribute to the clustering of prostate cancer grade and stage at diagnosis in Maryland.

There are some limitations in using surveillance data, such as the Maryland Cancer Registry data used here. Although population-level surveillance data are more comprehensive in terms of geographic and population coverage than clinical records from care settings, they are more likely to be missing clinical data of interest. Although the Maryland Cancer Registry has received the North American Association of Central Cancer Registries "gold standard" rating in most years, indicating the highest level of completeness and accuracy, in these data, grade and stage at diagnosis were missing for 17% and 16% of the registry population, respectively. Cases with missing grade or stage at diagnosis differed in age and location, though not race, from cases with complete diagnosis information [25]. Furthermore, in this analysis we used specific dichotomous cut-points for our two clinical outcomes, and it is likely that results would differ with other cut-points. For example, we have previously also examined these as ordinal outcomes [30].

More broadly, this analysis offers only one specific example with which to evaluate these tests. Next steps should include evaluations with data from other geographic areas and disease questions of interest. Although hot-spot detection of disease clusters remains a priority for clinically focused surveillance and medical services delivery, global tests of spatial randomness are also important tools for public health research.

## Competing interests

The authors declare that they have no competing interests.

## Authors' contributions

VH participated in the development of the study design, conducted parts of the analysis, participated in interpretation of the findings, and drafted the manuscript. AK obtained these data from the Maryland Cancer Registry, built and evaluated the multivariate models, created the datasets used in this analysis, and participated in the development of the analysis design and interpretation of the findings. CS worked with MK and VH to conduct parts of the analysis and assist with interpretation of the spatial analyses. MK obtained funding for this study, and was the PI on the project. He was responsible for conception and design of the study, and oversaw the analysis and interpretation of the findings. All authors contributed to and approved the final version of the manuscript.
